# P-1309. Analyzing the potential impact of 4CMenB and MeNZB vaccination strategies on gonorrhoea burdens in Singapore

**DOI:** 10.1093/ofid/ofae631.1490

**Published:** 2025-01-29

**Authors:** Lin Geng

**Affiliations:** Nanyang Technological University, Singapore, Not Applicable, Singapore

## Abstract

**Background:**

Gonorrhoea ranks among the top three most frequently reported sexually transmitted infections in Singapore. Evidence that the MeNZB and four-component serogroup B meningococcal (4CMenB) vaccines, designed against *Neisseria meningitidis*, can also offer partial cross-protection to satve Gonorrhoea transmission. However, how the efficacy and duration of protection, as well as the vaccination strategy affect a gonorrhoea vaccine’s impact to Singapore specific context have not been assessed.

**Figure 1:**
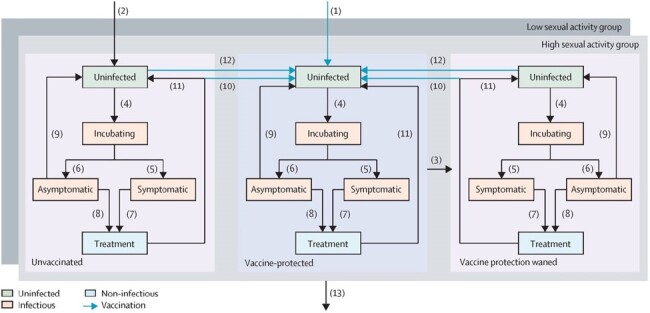
model structure diagram of gonorrhoea transmission in different vaccination status and sexual activity group The model patterned gonorrhoea infection stage into uninfected, incubating, infected (with possible of asymptomatic or symptomatic infectious) and treated stages. Certain level of population was set to be infected as gonorrhoea epidemiology seed while others remain uninfected. Those uninfected people who had sexual contact with infected people have a chance to get enough exposure of gonorrhoea bacteria and enter incubating compartment. People with gonorrhoea pathogen incubating in vivo will develop asymptomatic or symptomatic infection and become infectious to spread out gonorrhoea to uninfected population when they paired to have sexual contact. For those asymptomatic infection, patient may not notice any inflammation and recovered naturally. On the other hand, they may have a chance to attend sexual health clinic for regular STIs screening and tested out with gonorrhoea infection. The patients with symptomatic infection, we assume all of them seek treatment from sexual health clinic for their notifiable inflammation and burning sense. Both infection types get diagnosed when they have laboratory test positive and will receive anti-microbial as per entered pharmaceutical treatment compartment. The treated people become susceptible again to gonorrhoea because no effective immunological memory shall develop and function till next gonorrhoea infection. Given the pseudo vaccination protect individual against gonorrhoea offered to population by different strategy, the dimension of vaccination status compartments was created and asserted possible vaccination status including unvaccinated, vaccine-protected and vaccine protection waned, three disjoint scenarios. Given that MSM population has an imbalanced distribution on their sexual partner per year, we refined the variate gonorrhoea infection risks into high or low unit to better catering high or low sexual activity feature among population. Every individual exists solely in each dimension at a time and transferable at different time for every stage are mutually disjoint.

**Methods:**

We developed an integrated transmission-dynamic model, calibrated using Bayesian methods to local surveillance data to understand the potential population health impact of 4CmenB in reducing Gonorrhoea transmission. We explored the efficacy of implementating 3 specific vaccination programmes (1) offering vaccination to individuals attending to sexual health clincis for testing (vaccination on attendance [VoA]); (2) offering vaccination on individuals attending sexual health clinics and were diagnoses with Gonorrhoea (vaccination on diagnosis [VoD)]); or (3) vaccination according to risk (VaR), by offering vaccination to patients who were diagnosed with gonorrhoea plus individuals who tested negative but report having more than five sexual partners per year. We assessed efficacy by examining vaccination impact relative to the baseline case of no vaccination and during when behavioural parameters were held constant. We further ascertained the effects of varying vaccine uptake, vaccine efficacy and duration of protection.

**Figure 2:**
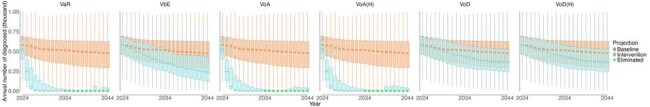
Total diagnosed future prediction in different vaccination strategy The series boxplot illustrated the future projection of thousand annual number of diagnosed of no vaccine baseline, different vaccination strategies as interventions for each column. The median of each box is marked in square shape and the 95% credible interval was drawn by the upper and lower box boundary. Certain exceptional great outliers were omitted for figure effect. The gonorrhoea incidence eliminated year is marked with green square indicating its median prediction of annual number of diagnosed falls below 1.

**Results:**

In a hypothetical 10-year vaccination programme, VoA had the protective impact on the population with 88.23% averted cases (95% CrI 3.79%-93.24%) but required more vaccine doses than any other strategy, VoD has a smaller impact (14.65% averted cases (95% CrI 2.27%-35.75%)) but requires only 1.67% total doses of VoA. VaR (88.13% averted cases (95% CrI 3.73%-93.22%)) had almost the same impact as VoA but required fewer doses administered than VoA. Sensitivity analyses indicated that VaR was the most balanced and impactful strategy for vaccines of any efficacy or duration of protection (or both).

**Figure 3:**
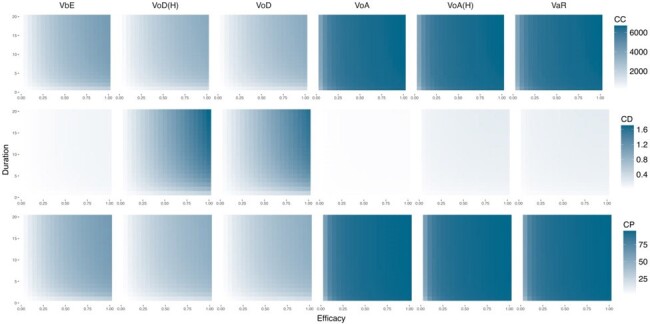
Sensitivity Analysis by varying vaccine profile

The series heatmap provided the impact change insight by varying vaccine duration of protection and efficacy in different strategy, evaluated in CC (k), CD and CP(%). The value is colored from light to dark indicating low to high. The light blue rectangle box drawn by the standard vaccine characters of duration equals 3 years and efficacy equals 31% to the maximum of each axis, suggesting an area where a vaccine in better performance would locate. The light blue plus marks were drawn by threshold suggesting a better performance compared to the best record achieved by standard vaccine in each strategy for each metric respectively.

**Conclusion:**

Vaccination of MSM against gonorrhoea according to risk in sexual health clinics in Singapore with the 4CMenB vaccine can be considered.

**Figure 4:**
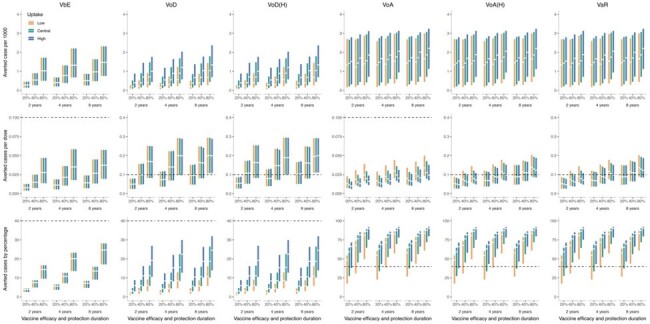
Multiple vaccine profiles - various uptake rates against gonorrhoea over 10 years The series of boxplot illustrate the vaccination strategy impact difference by varying vaccine uptake rates, vaccine duration of protection and vaccine efficacy. The uptake was set to be 0.5, 1.0 and 1.5 times of MSM HPV uptake rate in UK, and the vaccine quality scenarios were presented with pairs of 2, 4 and 8 years duration of protection with 20%, 40% and 80% of vaccine efficacy. The median of each box is marked in square shape and the 95% credible interval was drawn by the upper and lower box boundary. Certain exceptional great outliers were omitted for figure effect.

**Disclosures:**

**All Authors**: No reported disclosures

